# The Malaysian Northern Stars (supervision, training, and reflective system) project: a multi-facet ecosystem of producing local talents

**DOI:** 10.1192/bjo.2021.417

**Published:** 2021-06-18

**Authors:** Noor Melissa Nor Hadi, Jiann Lin Loo

**Affiliations:** 1 Hospital Tuanku Fauziah; 2 Ysbyty Maelor Wrecsam, Betsi Cadwaladr University Health Board

## Abstract

**Aims:**

The MRCPsych (Membership of the Royal College of Psychiatrists, United Kingdom) parallel training pathway has been introduced in Malaysia to produce competent psychiatrists to deliver evidence-based psychiatric care. Certain training centres faced specific challenges during the process of implementation, including the lacking of supervisors with experience in the MRCPsych examination, over-reliance on self-study and existing continuous medical education (CME), logistic difficulty in accessing specific training courses, the sustainability of local training, and loss of manpower due to frequent mobilisation of trainees. This article is aimed to illustrate the Northern STARS (Supervision, Training, and Reflective System) project, i.e. a project implemented as a solution for those challenges and an effort to develop a sustainable model of training for the local talents in Perlis, a northern state in Malaysia.

**Method:**

The Northern STARS initiatives included: setting up a library with more MRCPsych-related materials; introducing trainees to virtual MRCPsych support groups; organizing both physical and virtual training locally, collaborating with local and international experts for consultation and teaching, and the introduction of protected study time. Virtual platforms were used innovatively to minimise cost. Ongoing data were collected for programme evaluation and quality improvement. Trainees were actively involved in the process to facilitate the development of leadership and administrative skills.

**Result:**

A total of seven courses covering both skill and theory training had been organised: Ultra-brief Psychological Intervention Workshop, Dialectic Behavioural Therapy workshop, Personality Disorder Workshop, Critical Appraisal Workshop, MRCPsych Lecture Series, Addiction Psychiatry Lecture, and Basic Revision Course on Electroconvulsive Therapy. An estimated amount of twenty thousand Malaysian Ringgit had been generated and channelled into the community mental health centre, accounting for the indirect cost of a subscription to ZoomTM and the intangible cost of labour effort. Overall feedback revealed a high level of satisfaction together with some specific suggestions on areas of improvement, including the timing of course and coverage of the curriculum. To date, six medical officers are pursuing this pathway with three of them passing one paper and another two pursuing the final part.

**Conclusion:**

The Northern STARS project is an ecosystem of training solutions while generating income and producing more local talents to expand this project further. More long-term evaluation from the perspective of human resource and health economics can be considered to understand the efficiency of the current initiative.

